# Transient neonatal antibiotic exposure increases susceptibility to late-onset sepsis driven by microbiota-dependent suppression of type 3 innate lymphoid cells

**DOI:** 10.1038/s41598-020-69797-z

**Published:** 2020-07-31

**Authors:** Xinying Niu, Sarah Daniel, Dharmendra Kumar, Elizabeth Y. Ding, Rashmin C. Savani, Andrew Y. Koh, Julie Mirpuri

**Affiliations:** 1 Division of Neonatal-Perinatal Medicine, Department of Pediatrics, University of Texas Southwestern Medical Center, MC9063, 5323 Harry Hines Boulevard, Dallas, TX 75390-9063 USA; 2 Division of Hematology and Oncology, Department of Pediatrics, University of Texas Southwestern Medical Center, Dallas, TX USA

**Keywords:** Immunology, Infection, Mucosal immunology, Neonatal sepsis

## Abstract

Extended early antibiotic exposure in the neonatal intensive care unit is associated with an increased risk for the development of late-onset sepsis (LOS). However, few studies have examined the mechanisms involved. We sought to determine how the neonatal microbiome and intestinal immune response is altered by transient early empiric antibiotic exposure at birth. Neonatal mice were transiently exposed to broad-spectrum antibiotics from birth for either 3- (SE) or 7-days (LE) and were examined at 14-days-old. We found that mice exposed to either SE or LE showed persistent expansion of Proteobacteria (2 log difference, *P* < 0.01). Further, LE mice demonstrated baseline translocation of *E. coli* into the liver and spleen and were more susceptible *K. pneumoniae*-induced sepsis. LE mice had a significant and persistent decrease in type 3 innate lymphoid cells (ILC3) in the lamina propria. Reconstitution of the microbiome with mature microbiota by gavage in LE mice following antibiotic exposure resulted in an increase in ILC3 and partial rescue from LOS. We conclude that prolonged exposure to broad spectrum antibiotics in the neonatal period is associated with persistent alteration of the microbiome and innate immune response resulting in increased susceptibility to infection that may be partially rescued by microbiome reconstitution.

## Introduction

Seventy five percent of preterm babies admitted to the neonatal intensive care unit (NICU) receive antibiotics empirically^1,2^. Empiric antibiotics are generally broad-spectrum and prescribed based on risk assessment and not direct evidence of infection. The majority of infants in the NICU are exposed to transient antibiotics from birth that ranges from 2 to 7 days. Prolonged empiric antibiotics, lasting more than 2 days, are administered to approximately 35% to 50% of infants with a low gestational age^3^. Many babies born prematurely are also exposed to antibiotics in utero just prior to birth. Several clinical studies have demonstrated that exposure to transient early antibiotics in the NICU can increase the risk for development of late-onset sepsis (LOS)^1,2,4,5^. LOS is an important cause of morbidity and mortality in the NICU^5,6^.

Alteration of the intestinal microbiome and innate immune response may play an important role in increasing the risk for LOS in preterm infants. Early normal bacterial colonization is important for normal development of the gut^7^: strengthening and promoting gut barrier integrity^8,9^, protecting against pathogens^10^ and regulating host immunity^11–13^. Establishment of the microbiome during the neonatal period depends on multiple factors, including mode of delivery, breast versus formula feeding, gestational age, infant hospitalization, maternal diet and antibiotic use by the infant^14,15^. While antibiotics are beneficial for treating human bacterial infections, even short regimens can impact human gut microbial populations^16–18^ and prolonged broad-spectrum antibiotic therapy can significantly alter the normal balance of beneficial bacteria^19^.

The microbiome is also important in regulating immune responses in the intestine. Innate mechanisms of control are particularly crucial in the neonate^20^, when the adaptive immune response is still immature. Type 3 innate lymphoid cells (ILC3) are prominent in the fetal and neonatal intestine and produce the cytokines IL-17 and IL-22. IL-17 is important in host defense and IL-22 is crucial for barrier tissue regeneration and maintenance. The production of IL-17 by ILC3 is mediated by toll-like receptor (TLR) cytokine signaling. TLR signaling is important in the production of cytokines that expand ILC3, which produce IL-17 and have been implicated in neonatal LOS^21^. IL-17 increases the migration of neutrophils during infection, induces epithelial cells to produce antimicrobials, stimulates production of cytokines by macrophages and can increase mucus production by epithelial cells^22^. The role of ILC3 in neonatal host defense is emerging as crucial in mouse models and warrants further investigation.

In our studies described here, we hypothesized that transient antibiotic exposure in newborn mice would result in subsequent alteration of the microbiome and aberrant development of the innate immune response. To test this hypothesis, we exposed newborn mice to broad-spectrum antibiotics from birth for either 3- or 7-days, allowed mice to re-colonize and then subsequently analyzed the intestinal microbiome, cytokine profile of the small intestine and the presence of immune cells in the lamina propria at 14 days of life.

## Results

### Early empiric antibiotics induce a persistent alteration of intestinal microbial colonization in neonatal mice

**Figure 1:**
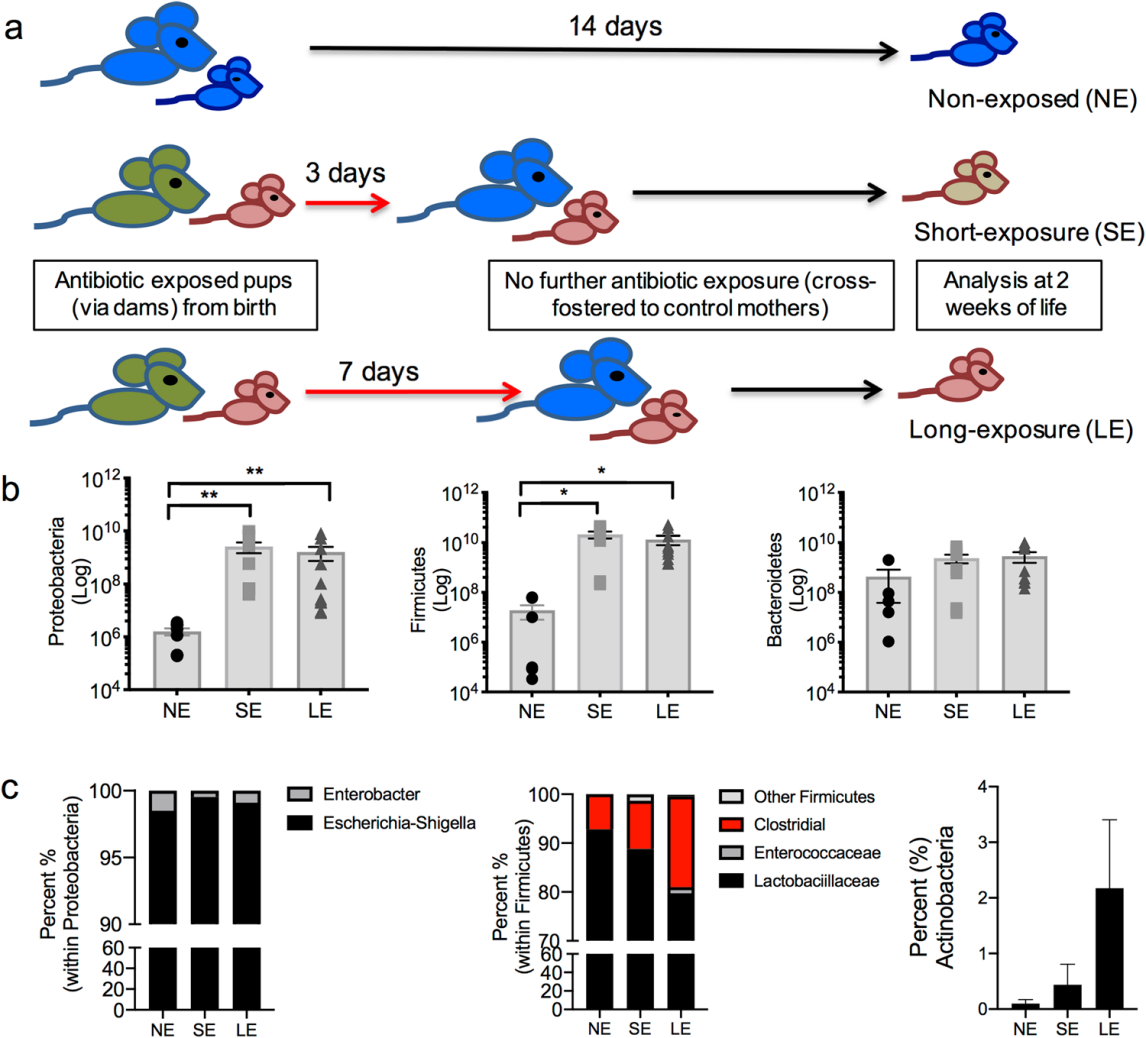
SE and LE mice had altered bacterial colonization at 2-weeks-old. (**a**) Experimental design. Control mice were born to mothers not exposed to antibiotics (blue mice) and sacrificed at 14 days of life (NE). Antibiotic exposed offspring were born to dams placed on broad-spectrum antibiotics (green mice) during gestation and offspring were exposed to antibiotics via their dams for either 3 days (SE) or 7 days (LE) by cross-fostering to mothers not on antibiotics (blue mice). All neonatal mice are analyzed at 2 weeks of life. (**b**) qRT-PCR of colonic fecal contents in neonatal mice for major bacterial phyla. SE and LE mice had an expansion of Proteobacteria and Firmicutes. (**c**) 16s rRNA sequencing identification of bacteria at the genus level reported within Proteobacteria and Firmicutes. Within Proteobacteria, LE mice had an increase in Escherichia. Within Firmicutes, LE mice had an increase in Clostridia. Actinobacteria was increased in LE mice. Data shown are representative of three experiments with a total of 6–10 mice in each group. Next-generation data are pooled samples (3 mice) of 3 mice/group. Data are depicted as mean ± SEM (*P < 0.05, **P < 0.01). Statistical tests used were ANOVA (**b**) and Kruskal–Wallis (**c**).

The neonatal period is a critical time for colonization of the intestine and even transient antibiotic exposure could disrupt the normal pattern of microbiome acquisition. In order to determine the effect of transient early neonatal antibiotics, we exposed newborn mice to either 3-days (SE, short exposure) or 7-days (LE, long exposure) of broad-spectrum antibiotics (ampicillin, vancomycin, neomycin, streptomycin and metronidazole) through their dams (Fig. 1a). Mice were then cross fostered to dams that were not exposed to antibiotics to allow them to recolonize. Controls were neonatal mice that were never exposed to antibiotics. All neonatal mice were then analyzed at 14 days of life (Fig. 1a).

In order to determine the effect on the intestinal microbiome, quantitative analysis of the major bacterial phylogenetic groups in colonic fecal contents was performed by qRT-PCR. We observed that, at 2 weeks of age, both SE and LE mice developed an expansion of γ-Proteobacteria and Firmicutes compared to non-exposed mice (NE) (Fig. 1b). Interestingly, we found no difference in the total number of bacteria in control, SE and LE mice (data not shown). We further qualitatively determined the composition at the genus level by utilizing next-generation sequencing identification of 16S rRNA from colonic fecal contents. In line with the qRT-PCR data, both SE and LE mice showed an increase in *Escherichia-Shigella (*within Proteobacteria), *Enterococcus* and *Clostridial spp*. (within Firmicutes) and in *Actinobacteria* in both the SE and LE mice, but with the LE mice showing the most difference compared to controls (Fig. 1c). Collectively, these data suggest that even transient antibiotic exposure at birth disrupts the normal pattern of colonization in neonatal mice.

### Extended neonatal antibiotic exposure promotes bacterial translocation at baseline and increases susceptibility to *K. pneumonia*e-induced sepsis at 2 weeks of life

**Figure 2:**
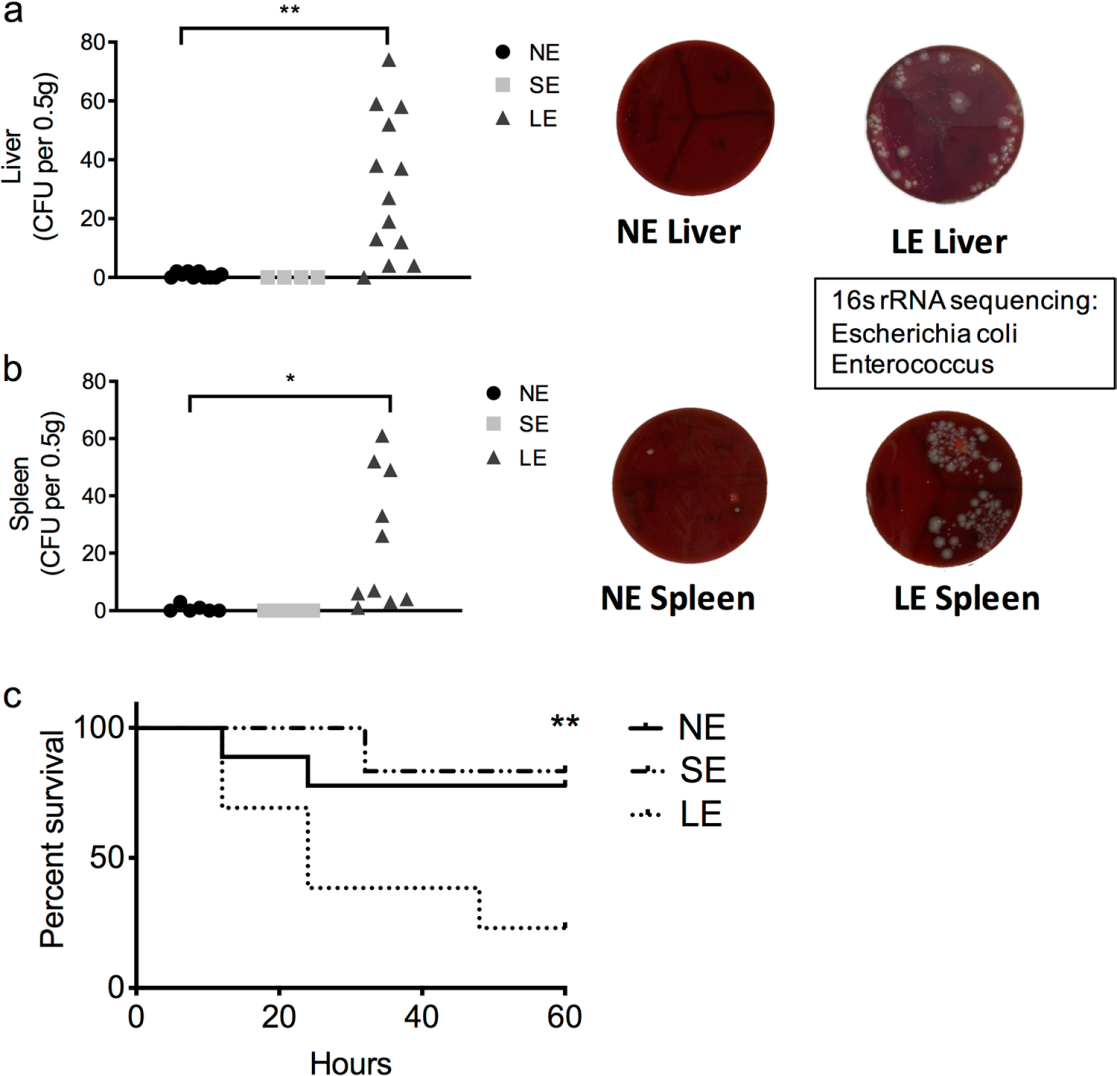
LE mice had baseline bacterial translocation to the liver and spleen and increased susceptibility to *K. pneumoniae sepsis*. (**a**) Baseline cultures from the liver of NE, SE and LE mice showing number of colonies grown on aerobic blood agar culture and representative culture plates from NE and LE mice. (**b**) Baseline cultures from the spleen of NE, SE and LE mice showing number of colonies grown on aerobic blood agar culture and representative culture plates on NE and LE mice. Anaerobic cultures did not grow any colonies (data not shown). Only the LE group had increased numbers of bacterial colonies. 16s rRNA sequencing of colonies grown from LE mice identified them as *Escherichia coli* and *Enterococcus*. (**c**) Kaplan–Meier survival graph of NE, SE and LE mice exposed to *K. pneumoniae* induced sepsis. Compared to the other groups, LE mice had significantly increased mortality. Data shown are representative of 4 experiments with 9–14 mice in each group. Statistical analysis used were ANOVA (**a**,**b**) and log-rank (**c**). Data are depicted as mean ± SEM (*P < 0.05, **P < 0.01).

In clinical studies, empiric antibiotic exposure at birth is associated with an increase in the incidence of late-onset sepsis^5^. We hypothesized that the altered microbiome in SE and LE mice is a source of bacteremia and late-onset sepsis. To test this, we collected blood, liver and spleen from NE, SE and LE mice and utilized aerobic and anaerobic culture to determine the presence of live bacteria. Interestingly, live bacteria were detected in the liver and spleen only in LE mice (Fig. 2a,b), but not in the blood (data not shown). Notably, utilizing 16S rRNA sequencing, we identified the bacteria seeding to the tissues as *Escherichia coli* and *Enterococcus*, both of which were increased in the colonic fecal contents of LE mice (Fig. 1c).

In order to determine if this increase in baseline translocation could result in susceptibility to LOS, we utilized an established neonatal murine mouse model for sepsis using intraperitoneal administration of *Klebsiella* pneumoniae (see “Methods” section). Indeed, we found that LE mice had increased susceptibility to sepsis with reduced survival when compared to NE and SE mice (25% vs 75%, P < 0.01, Fig. 2c).

### Extended transient neonatal antibiotic exposure decreased IL-17A, TLR-2 and TLR-4 expression in the intestine at 2 weeks of life

**Figure 3:**
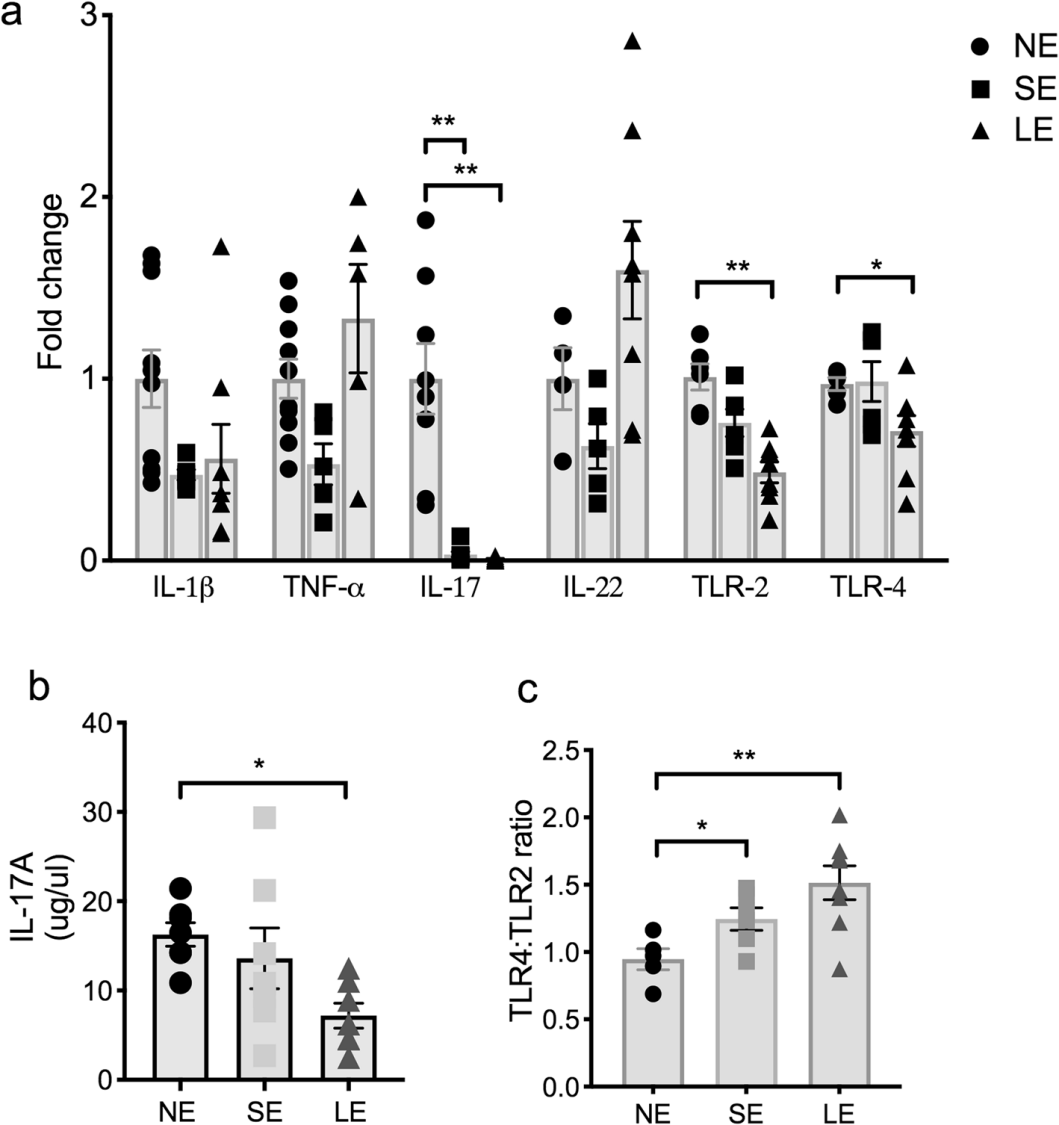
LE mice have decreased IL-17 cytokine production in the small intestine. (**a**) qRT-PCR of whole small intestine in NE, SE and LE mice demonstrated that LE mice had a significant decrease in IL-17A, TLR2 and TLR 4. (**b**) ELISA confirmed that LE mice had decreased production of IL-17A. The data shown are representative of 3 experiments with total of 6–10 mice in each group and analyzed with one-way ANOVA with post-hoc Tukey test. Data are depicted as mean ± SEM (*P < 0.05, **P < 0.01).

We further hypothesized that with the alteration of the microbiome in LE mice that there would be a change in TLR signaling and cytokine expression in the intestine. We therefore examined the expression of TLR2, TLR4, IL-1β, TNF-α, IL-17A and IL-22 in the small intestine. Inflammatory cytokine profiling of 2-week-old SE and LE mice showed that IL-17A expression was significantly decreased in the small intestine (Fig. 3a). This decrease in LE mice was confirmed by ELISA, which showed that IL-17A protein was decreased by almost 50% compared to NE mice (Fig. 3b). Interestingly, we found that the expression of TLR2 and TLR4 was also decreased in LE mice (Fig. 3a), with an increase in the ratio of TLR4 to TLR2 (Fig. 3c). TLR signaling is important in the production of cytokines that expand type 3 innate lymphoid cells (ILC3), which produce IL-17A and have been implicated in neonatal LOS^21^. This raises the possibility of decreased gram-negative bacteria mediated LPS-TLR4 signaling with extended early empiric antibiotic exposure.

### Extended transient neonatal antibiotic exposure results in suppression of IL-17A-producing ILC3 at 2 weeks of life

**Figure 4:**
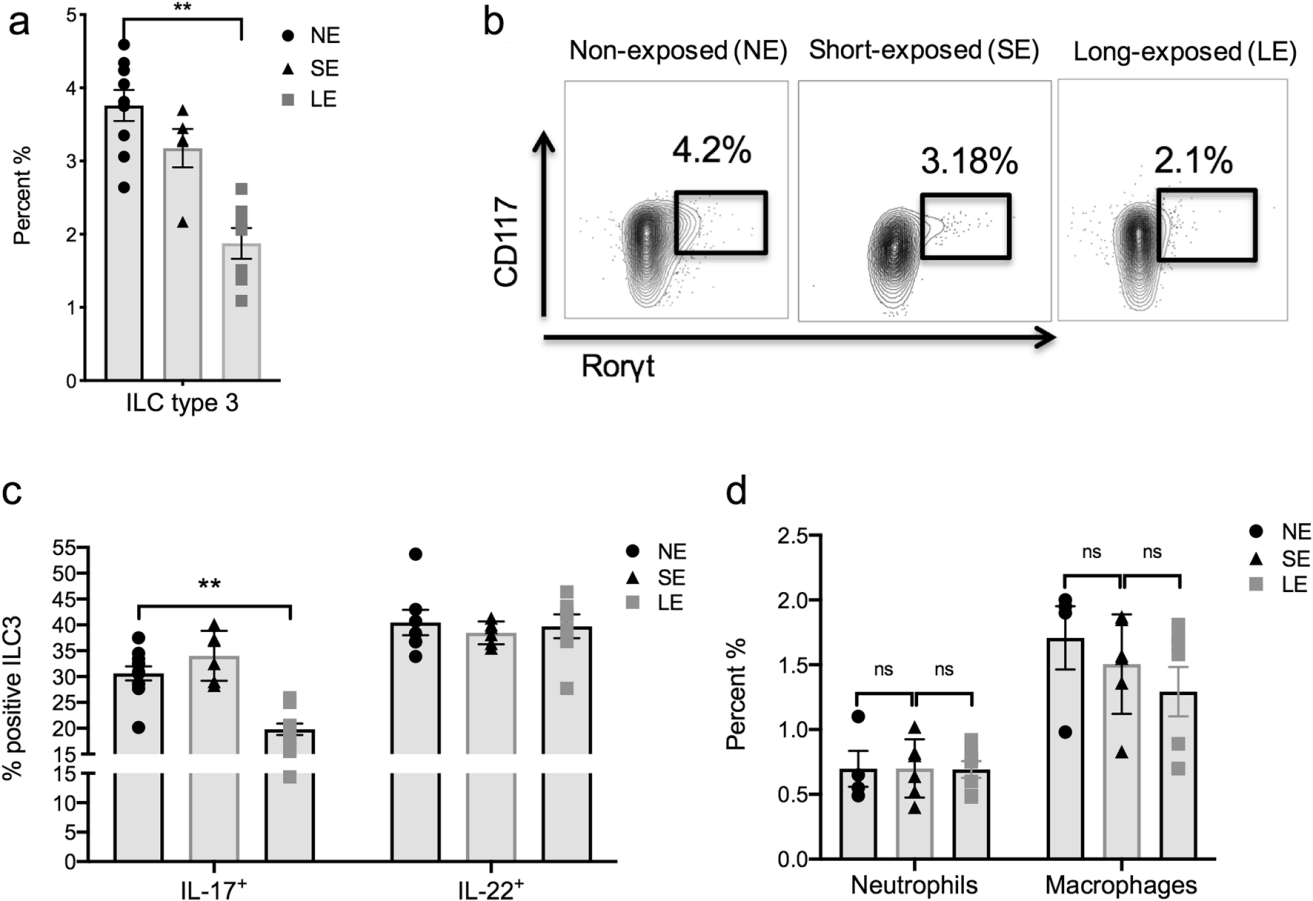
LE mice had suppression of IL-17A-producing type 3 innate lymphoid cells (ILC3). Flow cytometric profiling of small intestinal tissues of neonatal mice at 14 days of life treated with either SE or LE and compared to NE was performed. (**a**) The percentage of ILC3 (Rorγt^+^, CD127^+^, CD117^+^, CD3^–^, NKp46^+^, and CD4^–^) was lower in the LE group compared to NE and SE mice. (**b**) Representative flow cytometry panels showing percentage of ILC3 cells in SE, LE and NE mice. (**c**) By intracellular staining, the percentage of ILC3 that were positive for IL-17A were lower in LE mice, whereas there were no differences in IL-22 in any group. (**d**) Percentage of macrophages and neutrophils in SE, LE and NE groups showed no difference. Data are depicted as mean ± SEM (*P < 0.05, **P < 0.01) by one-way ANOVA with Tukey’s post-hoc test. The data shown are representative of 3 experiments with total of 5–8 samples in each group.

ILC3 are innate immune cells that have a dual role of defense against extracellular microbes as well as maintaining intestinal homeostasis^23^. ILC3 produce the cytokines IL-17A and IL-22. IL-17A is important in host defense in neonates^21^ and the presence of ILC3 is modulated by the microbiota^24^, including in neonatal mice^15^. We hypothesized that the presence of ILC3 would be decreased in LE mice that had increased susceptibility to our model of LOS. We performed flow cytometry to quantify the immune cells in the lamina propria in NE, SE and LE mice. We found ILC3, identified as Rorγt^+^, CD127^+^, CD117^+^, CD3^–^, NKp46^+^, and CD4^–^, were decreased in proportion compared to NE and SE mice at two weeks old (Fig. 4a,b). Further, intracellular staining for IL-17A showed fewer IL-17A producing ILC3 (Fig. 4c), whereas there were no differences in the representation of neutrophils or macrophages (Fig. 4d).

### Recolonization with mature microbiota after LE increases IL-17A-producing ILC3 and partially rescues mice from increased susceptibility to LOS

**Figure 5:**
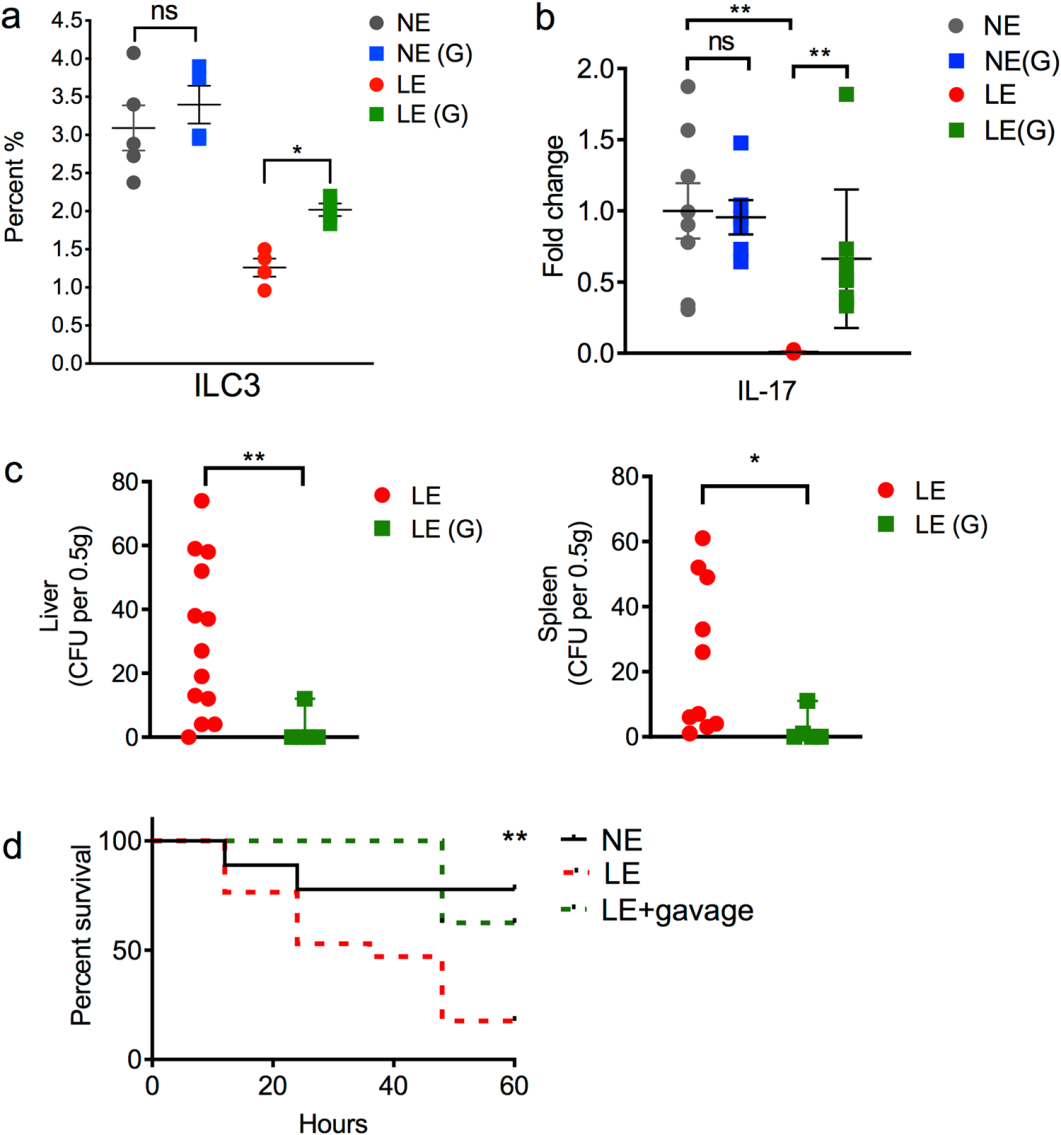
Re-colonization with mature microbiota after antibiotic exposure partially rescued the LE phenotype. After exposure to antibiotics, LE mice were re-colonized by gavage every other day with mature microbiota from 3-week old NE mice, depicted as LE(G). NE mice were exposed to mature microbiota or vehicle beginning at 7 days of life, depicted as NE(G). (**a**) Percentage of RORγt^+^ ILC3 NE and LE mice that were gavaged vehicle-control (circles) or gavaged (G) with mature microbiota (squares). LE(G) mice showed increased ILC3 compared to LE mice. (**b**) qRT-PCR for IL-17A in small intestine in NE, NE(G), LE and LE(G) mice. LE(G) mice had increased expression of IL-17A. (**c**) Aerobic culture of liver and spleen in LE and LE(G) mice/LE(G) mice did not have increased bacterial translocation at baseline. (**d**) Kaplan–Meier survival graph of NE, LE and LE(G) mice exposed to *K. pneumoniae*-induced sepsis. Mature microbiota-reconstituted LE mice had improved survival compared to non-reconstituted LE mice by log-rank test. Data are depicted as mean ± SEM (*P < 0.05, **P < 0.01). The data shown are representative of 3 experiments with total of 6–14 mice in each group.

Since we demonstrated that LE mice transitioned into an altered microbiota at 2 weeks of life, had an increased susceptibility to LOS and decreased IL-17A-producing ILC3, we next hypothesized that facilitating colonization with normal microbiota would rescue the LE-induced phenotype. We therefore re-colonized LE mice after exposure to antibiotics by gavage every other day with mature microbiome from 3-week old unexposed mice and examined them at 2 weeks of age. We found that reconstitution of the microbiota after antibiotic exposure in LE mice resulted in an increase in ILC3 (Fig. 5a). The expression of IL-17A in the small intestine was also increased (Fig. 5b). Further, bacterial translocation to the liver and spleen at baseline in LE mice that were recolonized was significantly diminished compared to LE mice that were not gavaged (Fig. 5c). Importantly, reconstitution of LE mice with mature microbiota was associated with a partial rescue from *K. pneumonia*-induced sepsis (Fig. 5d).

## Discussion

The aim of this study was to utilize an antibiotic exposure model to mimic a frequent scenario in the neonatal intensive care unit for preterm infants in order to mechanistically determine how early antibiotic exposure increases the risk for late-onset sepsis. In the present study, exposure of newborn mice to antibiotics for three or seven days was associated with an altered acquisition of the microbiome, with an expansion of γ-Proteobacteria and Firmicutes. In particular, with an extended antibiotic course (LE), this was accompanied by an overall decrease in IL-17A production in the intestine, an increase in baseline bacterial translocation and increased susceptibility to late onset sepsis. Importantly, LE was associated with a decrease in IL-17A-producing ILC3 at 2-weeks of life and re-colonization with a normal microbiome immediately after antibiotic exposure resulted in an increase in ILC3 and a partial rescue from late-onset sepsis.

Almost all preterm infants are exposed to antibiotics within the first few postnatal days^2^. Our model mimics the usual length of early antibiotic exposure in most NICUs, with antibiotics being started within 1 to 2 h after birth and the shortest courses lasting 48 h and the average length of treatment for culture-negative sepsis being 7 days. Most preterm infants are also exposed to antibiotics prenatally, with mothers being given prophylactic antibiotics for preterm labor and/or for rupture of membranes. Our study showed that with early transient use of antibiotics in newborns, the microbiome in the intestine was altered. It is interesting that only the LE mice showed baseline increase in bacterial translocation and an increased susceptibility to late-onset sepsis. Of note, the organisms that were present in the liver and spleen in LE mice, *Enterobacter* and *Enterococcus*, matched those that were increased in the intestine, implying that the intestine is the source of bacterial invasion, leading to LOS. A study by Taft et al. of infants that were followed longitudinally showed that, for infants with late onset sepsis with an organism identified, there were detectable levels of the organism in the stool samples in 82% of cases prior to disease onset^25^, also suggesting that the gut is the source of organisms contributing to sepsis.

Toll-like receptors play an important role in host defense and microbial colonization^26,27^. In our model, we found a decrease in the expression of both TLR2 and TLR4, which may be due to a decrease in bacteria during the antibiotic exposure since germ free mice have decreased expression of TLRs in the small intestine^28^. This could explain the greater decrease in TLR2 and TLR4 seen in LE mice. TLR signaling is also important in the production of cytokines that expand ILC3^21^, which may explain the decrease seen in ILC3. Interestingly, the TLR4 to TLR2 ratio was higher in antibiotic exposed mice. An increase in TLR4 is associated with increased gram-negative susceptibility^29,30^.

Type 3 innate lymphoid cells have only been described in the last 12 years and their importance and role in host defense are still under investigation^31^. We know that ILC3 are primarily present in the intestine, but have also been found in the skin and lung. They develop in utero and play an important role in development of inflammatory diseases in both adults^31^ and in the neonate^15,20,21^. The potential role of ILC3 in preterm infants is particularly intriguing since these innate cells are functioning in the absence of a mature adaptive immune response.

In a study by Deshmukh et al., IL-17A producing type 3 innate lymphoid cells were decreased with prolonged antibiotic exposure in neonatal mice^21^ and mediated by microbiota dependent suppression of neutrophils. While our findings of decreased IL-17A-producing ILC3 are similar to these observations, we did not observe any changes in neutrophils. This difference could be due to the transient exposure to antibiotics in our study that allowed the mice to recolonize. Both studies show that, after antibiotic exposure, while the suppression of IL-17A-producing ILC3 is persistent, recolonization with mature, normal microbiota after antibiotic exposure rescues mice from LOS. There is heterogeneity in ILC3^32^ in that they can produce both IL-17A and IL-22. The differential production of IL-17 and IL-22 by ILC3 seen in our study may be due to differential stimulation or signaling from cytokines^33^ and warrants further investigation. Indeed, ILC3 are emerging as an important component for both host and defense, and maintenance of epithelial integrity during the neonatal period^34^. Further work to investigate the presence and roles of ILC3 in human neonates is needed.

One significant drawback of the model is the use of multiple broad-spectrum antibiotics, since most NICU’s use a combination of two antibiotics for broad coverage of gram-positive and gram-negative organisms. However, of note, there is significant variation in practice^2^ and selecting 2 antibiotics would also not be representative. In our model, the exposure of neonatal mice to antibiotics is through their dams, which is effective in transferring to offspring. Cross-fostering further allows for cessation of antibiotic exposure and exposure of the offspring to an environment where they can re-colonize. It is also worth noting that antibiotic-exposed pups may also be affected by changes in maternal colonization and breast-milk composition due maternal antibiotic exposure. Early antibiotic exposure in juvenile mice is associated with in an increased risk for obesity^35^. Of note, we found no differences in offspring weight between antibiotic-exposed and control mice at time of sacrifice at 14 days of age.

Cross fostering could also contribute to changes in the microbiome or immune development. In initial experiments, we characterized control mice that were cross fostered at seven days of life and compared them to control mice that remained with their birth mothers. We found no difference in microbiota composition, cytokine profile or ILC3 by flow cytometry (data not shown), and therefore further studies were performed with control mice that remained with their birth mother. Further, we ensured that pups were effectively cross fostered by excluding pups that were not thriving at time of sacrifice or if cross fostered litter size was less than 30% from time of fostering.

In summary, this study supports a role for microbiota dependent suppression of IL-17A-producing ILC3 in increasing susceptibility to LOS. Further work to determine which bacterial species or bacterial products of metabolism are most critical is needed. Our study shows promise in developing microbial-based therapeutics to improve host-defense in newborn infants and subsequently improve morbidity and mortality.

## Methods

### Mice and empiric antibiotic exposure

All animal experiments were conducted according to the protocols approved by the UT Southwestern Medical Center Institutional Animal Care and Use Committee (IACUC). All experiments have been performed in accordance with the relevant guidelines and regulations outlined by the IACUC. C57BL/6 mice were used for all experiments and mice were obtained from the UT Southwestern Medical Center Mouse Breeding Core Facility and have been maintained for more than 2 generations within the same facility. Male and female mice were housed in the same cage for breeding in specific pathogen-free (SPF) conditions at animal facilities at UT Southwestern Medical Center in accordance with protocols approved by the respective Institutional Animal Care and Use Committees and were provided standard laboratory diet and water ad libitum. Antibiotic-exposed pregnant and nursing dams were exposed to broad spectrum antibiotics added to the drinking water: 1 g/L ampicillin, 0.5 g/L vancomycin, 1 g/L neomycin, 1 g/L Streptomycin and 1 g/L metronidazole, all obtained from Sigma. Neonatal mice received antibiotics through their dams. Short (SE) and long (LE) antibiotic exposure was accomplished by cross-fostering offspring either at 3 days or 7 days of life respectively to antibiotic naïve mothers. Antibiotic-exposed neonatal mice were compared to mice never exposed to antibiotics (NE). Cross-fostering success was confirmed by only analyzing mice that were cross-fostered with weight similar to control mice (approximately 6.5 g) and excluding litters that were reduced by more that 30% after cross fostering. All mice were sacrificed at 14 days of life for experimental analysis.

### Quantitative RT-PCR

This was performed as previously described^15^ as follows. Excised mouse intestine was homogenized and purified using TRIZOL reagent and subjected to first-strand cDNA synthesis by using iScript Reverse Transcription Supermix (Biorad). DNA from stool was extracted using ZR Fecal DNA miniprep (Zymo Research) and quantified using NanoDrop 2000c Spectrophotometer (Thermo Fisher Scientific). Real-time PCR was performed using SsoAdvanced Universal SYBR Green Supermix (Biorad) and the CFX Connect Real Time system (Biorad) according to the manufacturer’s instructions. Intestinal cytokines data were analyzed by the Ct threshold cycle method with normalization for starting template performed using a housekeeping gene, SRP-14. Bacterial abundance was determined using standard curves constructed with reference to DNA corresponding to a short segment of the 16s rRNA for E-coli that was amplified using conserved specific primers. Primer sequences were used as follows: Murine SRP-14 5′-AAGTGTCTGTTGAGAGCCACGGAT-3′ and 5′-CTGTCACTGTGCTGGTTTGCTCTT-3′; IL-17A 5′-TCCCTCTGTGATCTGGGAA-3′ and 5′-CTCGACCCTGAAAGTGAAGG-3′; IL-22 5′-CCC ATC AGC TCC CAC TGC-3′ and 5′-GGC ACC ACC TCC TGC ATA TA-3′; TNF-α 5′-CCACCACGCTCTTCTGTCTAC-3′ and 5′-TGGGCTACAGGCTTGTCACT-3′; IL-1β 5′-CCTTCCAGGATGAGGACATGA-3′ and 5′-TGAGTCACAGAGGATGG-GCTC-3′; TLR2 5′-GCCACCATTTCCACGGACT-3′ and 5′-GGCTTCCTCTTGGCCTGG-3′, TLR4 5′-TTTATTCAGAGCCGTTGGTG-3′ and 5′-CAGAGGATTGTCCTCCCATT-3′, Bacterial primers used are as follows: Eubacteria 5′-ACTCCTACGGGAGGCAGCAGT-3′ and 5′-ATTACCGCGGCTGCTGGC-3′; γ-Proteobacteria 5′-TAACGCTTGGGAATCTGCCTRTT-3′ and 5′-CATCTRTTAGCGCCAGGCCTTGC-3′; Enterobacteriaceae 5′-GTGCCAGCMGCCGCGGTAA-3′ and 5′-GCCTCAAGGGCACAACCTCCAAG-3′; Bacteroidetes 5′-GGTTCTGAGAGGAGGTCCC-3′ and 5′-GCTGCCTCCCGTAGGAGT-3′; Firmicutes 5′-GGAGYATGTGGTTTAATTCGAAGCA-3′ and 5′-AGCTGACGACAACCATGCAC-3′.

### Isolation of intestinal lamina propria (LP) cells and flow cytometry

Isolation of LP cells was performed as previously described^15^ and summarized as follows. The small intestine was removed and opened longitudinally, washed of fecal contents, cut into smaller sections and subjected to 2 sequential incubations in PBS with 0.5 M EDTA and 0.2 M DTT at 37 °C with agitation at 220 rpm to remove epithelial cells. The solution was discarded between incubation steps and replaced. The remaining tissue was agitated in PBS and then filtered through a strainer. The tissue was pat dried and minced and placed in the incubator for 30 min with gentle agitation at 110 rpm in 0.4 mg/mL of Collagenase D and 50 mg/mL of DNase I at 37 °C. The samples were then washed through a strainer (100 μm) and centrifuged at 1,200 rpm at 4 °C. LP cells were then washed with FACS buffer (PBS, 1% EDTA, 1% FBS) and stained with antibody cocktail for 20 min at 4 °C. The following antibodies were used (all from eBioscience unless otherwise noted): CD45- PE-Cy7 (30-F11), c-kit-APC (180627) (R&D Biosystems), NKp46–PerCP (29A1.4), CD127-PE-Cy7 (A7R34), CD8a-APC (53–6.7), CD45-FITC (30-F11) and CD4-FITC (GK1.5). Biotin conjugated CD3e (145-2C11), CD8a (53–6.7) and NKp46 (29A1.4) were obtained from Biolegend.

After extracellular staining, cells were fixed in IC fixation buffer (eBioscience), washed in permeabilization Buffer (eBioscience) and stained for intracellular antigens Rorγt-PE (AFKJS-9), IL-17-PE (eBio17B7), anti-mIL-17-PerCP (R&D Biosystems) and Biotin anti-mIL-22 (Biolegend) for 1.5 h at 4 °C. To obtain sufficient cells for analysis, 2 mice were pooled for LP prep per sample. Samples were read on FACSCanto (BD) and analyzed using FlowJo Software (TreeStar).

### Enzyme-linked immunosorbent assay (ELISA)

This was performed as previously described^15^ and briefly described as follows: Small intestine tissues from offspring were incubated overnight at 37 °C in cell culture media (RPMI). IL-17 concentration in the supernatant was quantified using ELISA (eBioscience) according to the manufacturer’s recommendations.

### 16s rRNA sequencing of bacteria

This was performed as previously described^15^ and summarized as follows: Colonic contents were extracted and genomic DNA isolated using ZR Fecal DNA miniprep (Zymo Research). 16S rRNA genes (variable region 4, V4) were amplified using a composite forward primer and a reverse primer containing a unique 10-base barcode that was used to tag PCR products from respective samples, as previously described^36^. The pooled products were sequenced using the Roche 454 Titanium (Roche, Basel, Switzerland) platform (University of Texas at Austin Genome Sequencing and Analysis Facility) using Roche/454 Titanium chemistry. 16S rRNA sequencing for P10 was performed amplifying the V4 region of the 16S rRNA gene using the same primers as described above but adapted for the Illumina (Illumina, San Diego, CA) platform (Illumina HiSeq 2000, University of Texas Southwestern Medical Center Genomics Core Facility, pair-end sequenced 100 bp reads).

Sequences were quality filtered and analyzed using the open source software package: Quantitative Insights Into Microbial Ecology (https://qiime.org/). 16S rRNA gene sequences were assigned to operational taxonomic units using UCLUST (https://drive5.com/usearch/manual/uclust_algo.html), with a threshold of 97% pair-wise identity, then classified taxonomically using Greengenes (https://greengenes.secondgenome.com/downloads). After resolving based upon these parameters, the percentage of each organism was individually analyzed for each sample providing relative abundance information within and among the individual samples based upon relative numbers of reads within each. Evaluations presented at each taxonomic level, including percentage compilations represent all sequences resolved to their primary identification or their closest relative.

### Aerobic and anaerobic blood agar culture

After decapitation euthanasia, the spleen and liver were removed aseptically and 0.5 g of each organ was homogenized in 0.5 mL of sterile PBS (Phosphate buffered saline) by using ceramic beads in Bead Ruptor 4 (Omni International) and 10 µL sample was plated onto blood agar plate and incubated at 37 °C for 48 h. For blood culture, the blood was obtained immediately after decapitation at 14 days of life. 10 µl blood was plated onto blood agar plate and incubated at 37 °C for 48 h. The bacterial load was expressed as the number of CFU per 10 mm of sample for blood and as the number of CFU per gram of organ. Blood agar colonies were enumerated and sub-cultured using nonselective medial (LB broth). The culture was allowed to grow for 24 h and 100 µL of this culture was inoculated again in LB broth. The culture was allowed to grow for a further 24 h followed by centrifugation for processing for 16s rRNA for next generation sequencing.

## Experimental design

### Colonization of LE mice with mature microbiota

Fecal colonic contents from 3-week old mice not exposed to antibiotics were pooled separately in PBS and homogenized for 60 s using Bead Rupter 4 (Omni International). Quantification of bacterial populations was performed by serial dilution on blood agar plates. LE mice were gavage fed 3-week-old colonic contents at 10^10^ CFUs every other day beginning at end of antibiotic course at 7 days of life. Mice were sacrificed at 2 weeks for flow cytometric analysis or exposed to *K. pneumoniae* sepsis model at 12 days of life. Two separate experiments were performed with a total of 4–8 mice in each group.

### *K. pneumoniae* sepsis model

*K. pneumoniae* (ATCC, 43816) was grown in Luria Bertani (LB) broth to log-phase growth (37 °C, 200 rpm). To mimic late-onset neonatal sepsis, we inoculated neonatal mice at post-natal day 12 with 10^5^ CFU g^−1^ *K. pneumoniae* via intraperitoneal (i.p.) injection. We examined mice twice daily for survival until 60 h of life. Baseline survival for NE mice was consistently between 75% and 80% at 60 h.

### Statistical analysis

Data were analyzed by one-way ANOVA with post-hoc Tukey test, two-way ANOVA, unpaired t-test or Kruskal–Wallis, using Prism (GraphPad Software, San Diego). The Kaplan–Meier log-rank test was used to compare survival between groups. Data were expressed as mean ± SE with significance defined as P value < 0.05. Two-sided student t-test was used for the comparison of two single groups and analysis of variance (ANOVA) for multiple group comparisons.
